# Genome Sequence of “*Caldinitratiruptor microaerophilus*,” a Unique Microaerophilic Bacterium Closely Related to *Symbiobacterium*

**DOI:** 10.1128/mra.01141-22

**Published:** 2023-01-19

**Authors:** Tatsuya Nishiyama, Riku Mukaiyama, Kenji Ueda

**Affiliations:** a Life Science Research Center, College of Bioresource Sciences, Nihon University, Kanagawa, Japan; University of Delaware College of Engineering

## Abstract

We present the genome sequence of “*Caldinitratiruptor microaerophilus*” JCM16183, isolated by Fardeau et al. from a French hot spring. This microaerophilic bacterium represents a novel taxon related to the genus *Symbiobacterium*. The high (71%) G+C content of its 3.60-Mb circular genome supports the divergence of this bacterium from *Clostridia*.

## ANNOUNCEMENT

The Gram-positive bacterium “*Caldinitratiruptor microaerophilus*” JCM16183 (1), which is a phylogenetically related to bacteria of the genus *Symbiobacterium* (2–4), was isolated from a French hot spring based on its microaerophilic and nitrate-reducing activity. These bacteria are currently classified into the class *Clostridia* of the phylum *Bacillota* (formerly *Firmicutes*) (5). However, a 16S rRNA gene-based phylogenetic analysis performed by Watanabe et al. based on the identification of Limnochorda pilosa (6, 7) inferred their affiliation with a novel taxonomic group distinct from *Clostridia.* Here, we studied the entire genome sequence of *C. microaerophilus* to increase our knowledge of Gram-positive bacteria.

Freeze-dried stocks of *C. microaerophilus* JCM16183 cells purchased from JCM, RIKEN (Tsukuba, Japan), were revived and cultured anaerobically in a 300-mL closed glass vessel containing 250 mL liquid medium. The composition of the medium and anaerobic gas conditions (N_2_/CO_2_ ratio, 80%:20%, vol/vol) were described previously (1). The inoculum was incubated without shaking at 60°C for 48 h.

Genomic DNA was isolated from 0.2 g (wet weight) cells using the MagAttract high-molecular-weight (HMW) DNA kit (Qiagen). The DNA quality was assessed using a 5200 fragment analyzer system and a high-sensitivity (HS) genomic DNA 50-kb kit (Agilent). The genomic DNA of JCM16183 was then sheared to an average size of 10 to 20 kb using gTUBE devices (Covaris). A genomic library was constructed using a SMRTbell Express template prep kit v2.0 (Pacific Biosciences). After the formation of the DNA library-polymerase complex using the PacBio binding kit v2.2, a 10- to 20-kb library of the JCM16183 genome was sequenced using a PacBio IIe sequencer.

For the following data processing, default parameters were used for all software. Adapter sequences and short reads were eliminated using SMRT Link v10.1.0.119528 and Filtlong v2.0, respectively. The *de novo* assembly of 33,248 PacBio subreads (*N*_50_, 6,454 bp), performed using Flye v2.8.3, successfully produced a single closed contig. The reliability and completeness were calculated using Bandage v0.8.1 and CheckM v1.1.2, respectively. Assembly of the data generated a closed contig, and the circularized genome sequence was subjected to annotation using DFAST (8).

The single contig consisted of 3,599,694 bp, with a G+C content of 71.0%. The sequence assembly did not contain any plasmids. Using DFAST, 3,544 protein-coding sequences (CDSs), 12 rRNAs, and 69 tRNA genes were predicted. Similarly, as previously observed with the genome of Symbiobacterium thermophilum (4), JCM16183 harbored many CDSs for amino acid ABC transporter, 18 CDSs for RNA polymerase sigma factor, and those encoding proteins involved in endospore formation. The organism also retained CDSs for the cytochrome oxidase complex but did not retain any genes for catalase. The high G+C content and the presence of genes for aerobic respiration support the view that JCM16183 is distinct from *Clostridia*.

### Data availability.

The conditions used for the cultivation of JCM16183 are available at GenBank under BioProject accession no. PRJDB13120 and BioSample accession no. SAMD00445401. The complete genome sequence of JCM16183 has been deposited at DDBJ/GenBank under accession no. AP025628. We submitted the raw sequencing reads to the DRA under accession no. DRR414026.
